# Combining Oncolytic Adenovirus with Radiation—A Paradigm for the Future of Radiosensitization

**DOI:** 10.3389/fonc.2017.00153

**Published:** 2017-07-24

**Authors:** Sean M. O’Cathail, Tzveta D. Pokrovska, Timothy S. Maughan, Kerry D. Fisher, Leonard W. Seymour, Maria A. Hawkins

**Affiliations:** ^1^Cancer Research UK/Medical Research Council Oxford Institute for Radiation Oncology, Department of Oncology, University of Oxford, Oxford, United Kingdom; ^2^Department of Oncology, University of Oxford, Oxford, United Kingdom

**Keywords:** oncolytic virus, radiation, radiosensitizer, adenovirus, immunotherapy

## Abstract

Oncolytic viruses and radiotherapy represent two diverse areas of cancer therapy, utilizing quite different treatment modalities and with non-overlapping cytotoxicity profiles. It is, therefore, an intriguing possibility to consider that oncolytic (“cancer-killing”) viruses may act as cancer-selective radiosensitizers, enhancing the therapeutic consequences of radiation treatment on tumors while exerting minimal effects on normal tissue. There is a solid mechanistic basis for this potential synergy, with many viruses having developed strategies to inhibit cellular DNA repair pathways in order to protect themselves, during genome replication, from unwanted interference by cell processes that are normally triggered by DNA damage. Exploiting these abilities to inhibit cellular DNA repair following damage by therapeutic irradiation may well augment the anticancer potency of the approach. In this review, we focus on oncolytic adenovirus, the most widely developed and best understood oncolytic virus, and explore its various mechanisms for modulating cellular DNA repair pathways. The most obvious effects of the various adenovirus serotypes are to interfere with activity of the MRE11-Rad50-Nbs1 complex, temporally one of the first sensors of double-stranded DNA damage, and inhibition of DNA ligase IV, a central repair enzyme for healing double-stranded breaks by non-homologous end joining (NHEJ). There have been several preclinical and clinical studies of this approach and we assess the current state of progress. In addition, oncolytic viruses provide the option to promote a localized proinflammatory response, both by mediating immunogenic death of cancer cells by oncosis and also by encoding and expressing proinflammatory biologics within the tumor microenvironment. Both of these approaches provide exciting potential to augment the known immunological consequences of radiotherapy, aiming to develop systems capable of creating a systemic anticancer immune response following localized tumor treatment.

## Introduction

Radiation therapy is responsible for an estimated 40% of all the cured cancers worldwide (1). Modern radiotherapy techniques allow for reduced toxicity due to improved accuracy and modulation of radiation delivery. The therapeutic window between efficacy and toxicity is often optimized by the addition of a radiosensitizer. However, the development of novel radiosensitizers is challenging, with cytotoxic chemotherapy remaining the mainstay of radiosensitization in most solid organ tumors. There is an unmet need for rational development of radiation–drug combinations, as a relatively modest change in therapeutic index could have significant implications regarding improving outcomes. Apart from the addition of cetuximab to radical head and neck radiotherapy (2), no other targeted agent has been approved for radiosensitization in the last decade. Better understanding of the biological effects of radiation at a molecular level and the expanding availability of drugs that act on the specific pathways of radiobiological damage offers new opportunities. The unique nature of radiation injury and its cellular damage means that it is ideally suited for combination with oncolytic viruses. Here, we will discuss the nature of both radiation-induced DNA damage and the interaction between oncolytic adenoviral proteins and the DNA damage response, the behavior of oncolytic viruses in a cancer context and the proposed mechanism of synergy gained from their combination. We will also summarize the preclinical and clinical data to date, including toxicity therein, the role of the immune system in optimizing the effectiveness of combination therapy and, finally, the ability to arm oncolytic viruses to maximally contribute to effective synergy.

## Radiation-Induced DNA Damage and Repair

**Figure 1:**
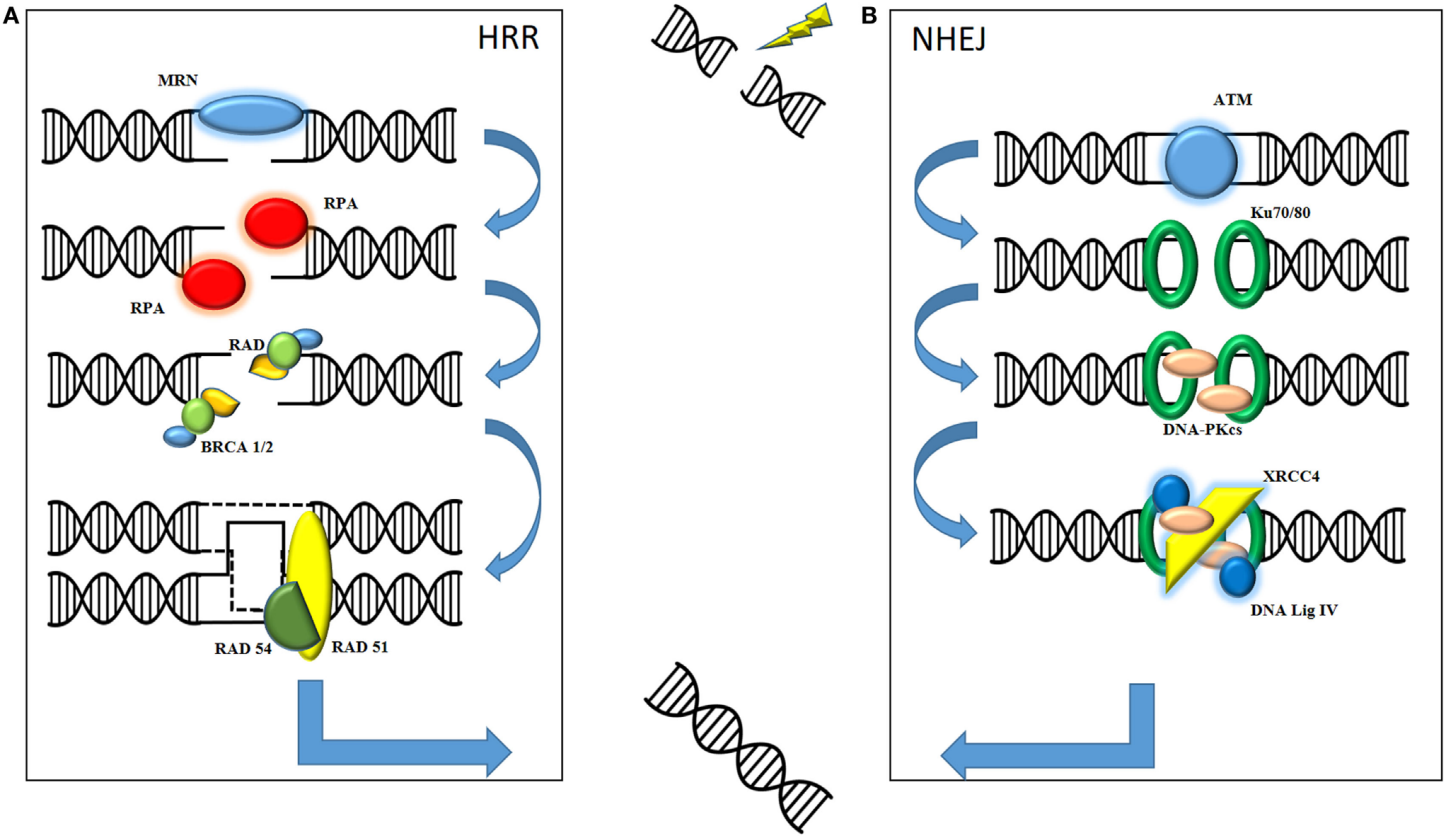
Ionizing radiation causes fatal double-strand breaks. DNA damage repair is mediated by two main pathways: homologous recombination repair (HRR) and non-homologous end joining (NHEJ). **(A)** In HRR, damage is sensed by the MRE11–Rad50–Nbs1 (MRN) complex, consisting of MRE11, Rad50, and Nbs1, which facilitates recruitment of downstream mediators to the site of damage. These include replication protein A (RPA), the Rad family of proteins and BRCA1 and BRCA2. Final sequence homology for the damaged DNA is provided by invading, and requires the presence of, the sister chromatid. **(B)** NHEJ is initiated by the recruitment of phosphatidylinositol-3-kinase-related kinase (PIKK) family such as ataxia-telangiectasia mutated (ATM). These facilitate the recognition of damaged strands by Ku70/Ku80, subsequent processing by DNA-PKcs and final repair and processing of strand ends by XRCC4 and DNA Ligase IV. The final product of both pathways is a repaired, complete strand of DNA.

The therapeutic effect of radiation on cell kill is mediated through DNA damage, specifically double-strand DNA damage, resulting in irretrievable cell death. Here following is a basic overview of the two main types of DNA damage produced by ionizing radiation (see Figure 1). It is important to understand the role of both non-homologous end joining (NHEJ) and homologous recombination repair (HRR) in order to understand how the combination with oncolytic virus may prove useful in the future. NHEJ is classically described as occurring in the G1 phase of cell division when there is no sister chromatid to act as a template. HRR is described mainly in G2/S phase when the presence of an undamaged chromatid acts as a template for repair of the affected DNA strand. Although the processes are not mutually exclusive, a cursory separation allows for an informed mechanistic explanation. The role of the MRE11–Rad50–Nbs1 (MRN) complex appears to be central to both forms of repair as a sensor for double-strand breaks (3).

### Non-Homologous End Joining

Protein kinases belonging to the phosphatidylinositol-3-kinase-related kinase (PIKK) family, such as ataxia-telangiectasia mutated (ATM) and ATR, are recruited to the site of DNA damage. Recognition of the damaged strands is facilitated by Ku70/Ku80. Subsequent recruitment of DNA-dependent protein kinase catalytic subunit (DNA-PKcs), which autophosphorylates to allow release from the DNA, means that end processing and ligation of the strands can commence. This is primarily mediated by DNA ligase IV and X-ray repair cross-complementing group (XRCC4). Consequently, these often form the basis for translational work, with their presence indicating significant DNA damage in need of repair.

### Homologous Recombination Repair

In contrast to NHEJ, HRR is a high fidelity repair mechanism for mammalian DNA. The double-strand break is sensed by the MRN complex, which processes the DNA into 3′ DNA single strands. The MRN complex allows for recruitment and activation of ATM, a key regulator of HRR. Autophosphorylation of ATM allows for downstream recruitment of repair proteins, such as BRCA1 and BRCA2, replication protein A (RPA), and other mediators such as the Rad family of proteins (4). Rad51 is a key protein as it mediates the invasion of the sister chromatid of the homologous strand to allow for accurate replication of DNA. The 3′ end of the ssDNA invades the homologous strand of the sister chromatid to form a “displacement” loop and the sequence is then extended by synthesizing new DNA to form a Holliday junction. Gap filling occurs by DNA synthesis beyond the original break site before Rad54 facilitates release of the newly synthesized end (5). The DNA strand is then annealed with the other end of the ssDNA to complete the repair process.

Although these two pathways are key mediators of the DNA damage response pathway, it has become clear recently that post translational modifications have a key role to play in coordinating the cell’s repair (6). This can include modification of the proteins themselves, phosphorylation of cyclin dependent kinases to control cell phase, ubiquitylation and sumoylation, and the regulation of checkpoints.

## Oncolytic Adenoviruses and the DNA Damage Response

### The Concept of Virotherapy

Oncolytic virotherapy uses lytic viruses that replicate selectively within cancer cells. This approach combines targeted cytotoxicity with amplification of the therapeutic agent actually within tumor cells. With the first Federal Drugs Administration (FDA) approval of an oncolytic virus, Talimogene laherparepvec (T-vec) in 2015 (7), interest in oncolytic virotherapy is rapidly expanding. This is, however, a field that has been developing for many years (8) and a wealth of information is now available on the ways in which these viruses can be incorporated in the current anticancer therapeutic paradigms (9).

A range of oncolytic viruses are currently undergoing clinical trials, including adenovirus, vaccinia virus, herpes simplex virus (HSV), reovirus, poxvirus, coxsackievirus, Newcastle disease virus, measles virus, parvovirus, Seneca Valley Virus, poliovirus, and vesicular stomatitis virus (10). The mechanism through which these viruses lead to tumor cell death varies between types, however it is the ability of certain oncolytic viruses to interact with and inhibit the DNA damage response which is of particular interest with regard to combining treatment with radiotherapy (11). Nuclear-living DNA viruses might be particularly sensitive to cellular DNA repair mechanisms and, therefore, could have developed strategies to interact with these cellular factors to protect viral DNA from unwanted repair (12).

**Figure 2:**
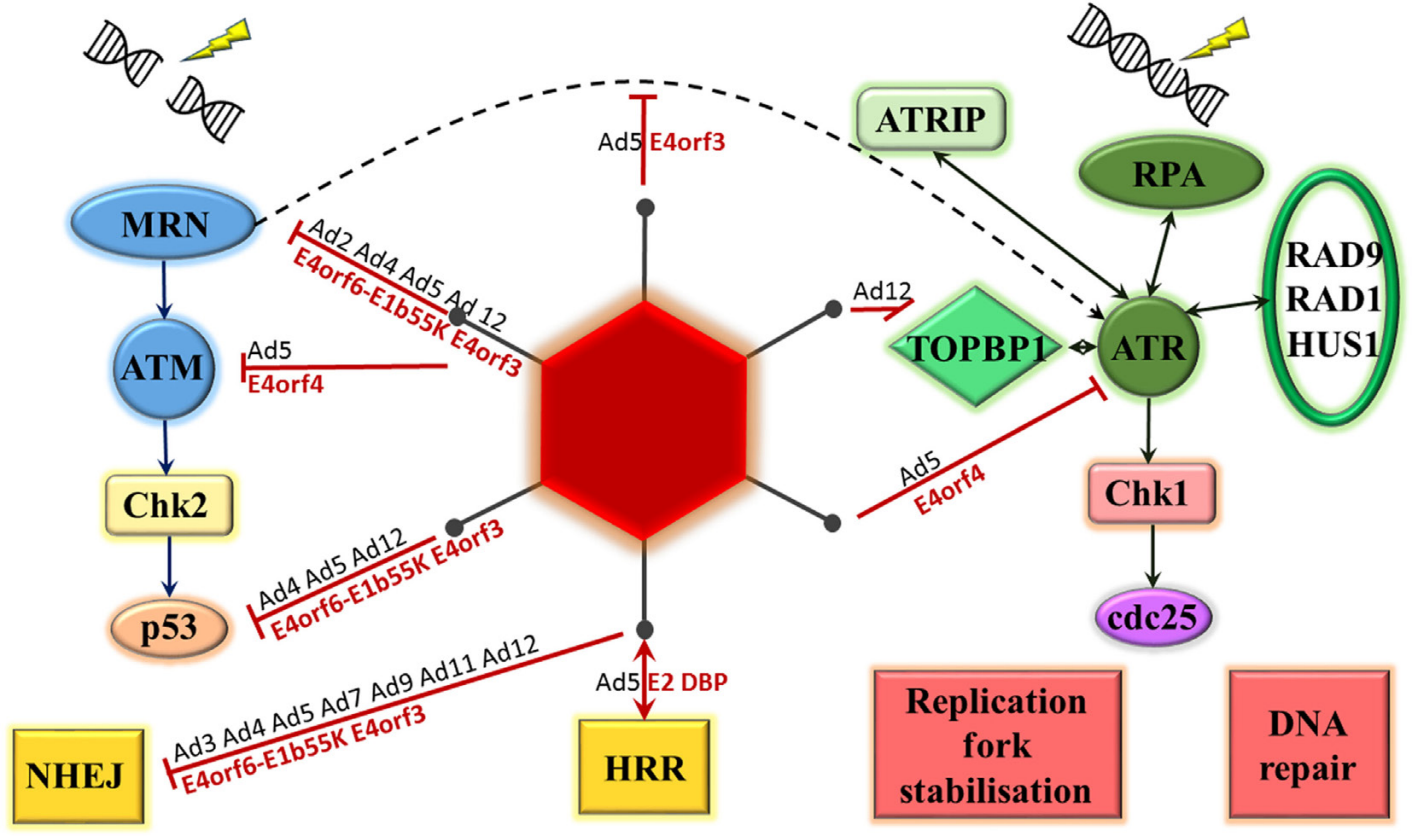
Adenovirus proteins interact with the DNA damage response. Double-strand breaks result in MRE11–Rad50–Nbs1 (MRN) complex activation of ataxia-telangiectasia mutated (ATM). This leads to phosphorylation of checkpoint kinase 2 (Chk2), activation of p53 and DNA damage repair through either non-homologous end joining (NHEJ) or homologous recombination repair (HRR). Single-stranded DNA is bound by replication protein A (RPA), which recruits ATM and RAD3-related (ATR) kinase, ATR-interacting protein (ATRIP), RAD9-RAD1-HUS1 and topoisomerase-IIβ-binding protein 1 (TOPBP1) to site. ATR phosphorylates checkpoint kinase 1 (Chk1) resulting in phosphorylation of the cell division cycle 25 (cdc25) phosphatases and a number of cellular changes, including DNA repair, effects on cell cycle, and stabilization of replication forks. Adenoviral proteins interact with a number of these steps, the most studied is adenovirus 5. Serotype of interacting adenovirus (Ad) denoted in black, adenovirus 5 protein identified as mediating interaction denoted in red.

There is considerable evidence available on the ability of adenoviruses, in particular, to inhibit the DNA damage response as part of the normal virus life cycle (see Figure 2). It is hypothesized that this is to prevent the linear double-stranded DNA viral genome from being recognized by the cell as a double-stranded DNA break, potentially leading to concatemerization of virus genomes (13, 14). This was first observed using a range of mutant adenovirus type 5 and type 2 on KB cells, a human epidermoid cell line. The mutations were all located in the early region 4 (E4) region of the genome and, when both E4orf3 and E4orf6 regions were mutated, concatemerization of the viral genome was noted (15).

### The Effect of Virus Proteins on the MRN Complex

The key cellular factor involved in sensing double-stranded DNA damage, particularly in the HRR pathway, is the MRN complex. The majority of the evidence relating to the interaction between adenoviral proteins and the DNA damage response is consistent with an effect on this complex. This was initially demonstrated by Stracker et al (16). Adenoviral genome concatemers formed in cells infected with adenovirus serotype 5 (Ad5) lacking the E4 region, but not with wild-type virus. However, no concatemers formed following infection with this mutant virus in cell lines containing any of mutant DNA ligase IV, DNA-PKcs, Nbs1, or MRE11, suggesting that these cellular proteins are all required for recognition and concatemerization of the adenoviral genome. In addition, in cells infected with the E4 mutant virus, the MRN complex could be seen to form foci surrounding viral replication centers. This paper was the first to provide evidence for MRN degradation mediated by the Ad5 E4orf6–E1b55K complex and MRN mislocalization mediated by Ad5 E4orf3 (16). These two mechanisms of MRN inhibition will be briefly explored below.

#### Degradation of the MRN Complex

Stracker et al. initially demonstrated that intracellular levels of MRE11, Rad50, and Nbs1 decreased following infection with wild-type adenovirus serotype 5 (Ad5). This was due to enhanced protein turnover and was not seen following infection with E4-deficient Ad5. Infection with Ad5 mutants lacking different E4 genes demonstrated that there was no degradation of MRE11 and Rad50 when cells were infected with Ad5 specifically lacking E4orf6–E1b55K. Furthermore, transfection of 293 cells with an E4orf6 expression vector resulted in MRE11 and Rad50 degradation, but not transfection with an expression vector containing mutant E4orf6 unable to form a complex with E1b55K. These, therefore, demonstrated that MRN degradation following infection with Ad5 is mediated by the E4orf6–E1b55K complex (16). Karen et al. further showed that this MRN degradation occurs prior to viral DNA accumulation (14).

The majority of evidence available is on the function of Ad5 proteins. Stracker et al. compared effects on MRN following infection with Ad5, Ad4, and Ad12. Infection of HeLa cells with all three serotypes led to decreased levels of MRE11 and Rad50. Likewise, transfection of 293 cells, which stably express Ad5 E1b55K, with plasmids encoding E4orf6 from these serotypes led to degradation of all MRN components as well as p53. These results suggest that the ability of the E1b55K–E4orf6 complex to cause MRN degradation is conserved between these serotypes (17). Forrester et al. have also demonstrated MRE11 degradation following infection with Ad4, Ad5, and Ad12, but not following infection with Ad3, Ad7, Ad9, and Ad11 (18). Therefore, although MRN degradation does appear to be targeted by a range of adenovirus serotypes, this is not conserved across all serotypes.

#### Relocalization of the MRN Complex

Stracker et al. demonstrated that early after infection by adenovirus serotype 5 there appears to be relocalization of the MRN complex to areas of nuclear speckles partially overlapping with promyelocytic leukemia protein (PML) (16). In uninfected cells PML is found in oncogenic domains (PODs/ND10), however Ad5 E4orf3 causes relocalization of PML into nuclear tracks (16, 19, 20). This function appears to be conserved across serotypes and has been demonstrated for Ad2, Ad4, and Ad12 E4orf3 (17, 20).

Stracker et al. further showed that this MRN relocalization does not occur following infection with Ad5 lacking E4orf1 to E4orf3. Transfection of 293 cells with different expression vectors showed that expression of E4orf3 is sufficient for relocalization of the MRN complex to occur. The relocalization of MRN can, therefore, be linked directly to expression of Ad5 E4orf3 (16).

It has since been demonstrated that at later time points of infection Ad5 E4orf3 causes redistribution of the MRN complex to large single juxtanuclear cytoplasmic accumulations suggestive of aggresomes. These contain both E4orf3 and E1b55K, but seem to be able to form in the absence of the latter (21, 22). Aggresomes are areas of ubiquitin-rich cytoplasmic inclusions containing aggregated misfolded proteins and surrounded by a cage of the intermediate filament protein vimentin. They are proposed to be a cellular response to undegraded, aggregated protein (23) and their formation has been linked to a number of degenerative diseases (24). Infection of A549 cells with an E4orf6/E4orf3 mutant Ad5 has been shown to greatly delay both aggresome formation and MRE11 localization to these areas, but not infection with Ad5 lacking only one of these viral proteins. Therefore, both Ad5 E4orf3 and E4orf6 can be seen to be key proteins involved in MRE11 relocalization during adenovirus infection, and appear to be functionally redundant (22). This relocalization to the cytoplasm accelerates degradation, thereby acting to protect the viral genome from recognition by the MRN complex and subsequent concatemerization (22).

Evidence provided by Liu et al. suggests that E1b55K also plays an important role in the formation of MRN-containing cytoplasmic aggresomes. They utilized leptomycin B to inhibit an exportin interacting with an E1b55K nuclear export signal. In cells infected with an Ad5 E4orf6 deletion mutant, which allows visualization of MRN without its degradation, leptomycin B caused inhibition of MRE11 relocalization from the nucleus to juxtanuclear aggresomes. Furthermore, addition of leptomycin B to wild-type Ad5-infected cells resulted in a decreased rate of MRE11 depletion. Finally, there was minimal exportation of MRE11 out of the nucleus following infection of cells with either an E1b55K deletion mutant Ad5 or Ad5 containing mutant E1b55K unable to bind MRN. These results suggest that E1b55K plays an important role in the relocalization and degradation of the MRN complex following infection with Ad5 (22).

Interestingly, when comparing the effects of Ad5 E4orf3 with that of Ad4 and Ad12, Stracker et al. found that, though Nbs1 appeared to be relocated away from viral replication centers following infection with Ad5, Ad4 and Ad12 did not have the same effect. These results would suggest that there are some differing effects on the relocalization of MRN components by different viral serotypes (17). Results published by Forrester et al., on the other hand, demonstrated that Ad5, Ad9, and Ad4 infection caused MRE11 relocalization to PML tracks. Infection with Ad3, Ad7, Ad11, and Ad12 resulted in relocalization of MRE11 to viral replication centers, as demonstrated by areas of RPA32 staining (18). These results are consistent with the view that different virus serotypes have varying effects on the MRN complex, and demonstrate some interesting differences in the results pertaining to Ad4 effects on members of the MRN complex.

### The Effects of Virus Proteins on Phosphatidylinositol-3-Kinase-Related Kinase (PIKK) Family

Ataxia-telangiectasia mutated and ATR play pivotal roles in the cellular response to DNA damage. Brestovitsky et al. have recently presented some data on the role of Ad5 E4orf4 in inhibiting the DNA damage response through effects on these proteins and their substrates. Comparison of the phosphorylation status between cells infected with an Ad5 E4 deletion mutant and an Ad5 E4 deletion mutant expressing only E4orf4 was carried out. Addition of E4orf4 significantly decreased the levels of phosphorylation of all ATM and ATR pathway proteins tested. This was shown to be dependent on E4orf4 interaction with the cellular protein phosphatase 2A (PP2A), which is known to dephosphorylate a number of proteins involved in the DDR including ATR, ATM, DNA-PK, and a range of their substrates. Both the cytopathic effect of E4orf4 and viral replication were significantly enhanced in cells lacking wild-type ATM and treated with an ATR inhibitor. Lack of wild-type ATM appeared to be beneficial during the early part of the virus life cycle and ATR inhibition during the late part of the virus life cycle. Moreover, expression of E4orf4 alone in cells was sufficient to inhibit DNA damage repair and to sensitize cells to the effects of genotoxic drugs even out with the context of infection (25). These results suggest inhibition of ATM and ATR is beneficial for the Ad5 viral life cycle and is mediated via an interaction between E4orf4 and PP2A.

#### The Effects of Virus Proteins on ATM

It has been suggested that, in the context of infection with Ad5 mutant viruses lacking E4 and unable to interfere with the MRN complex, ATM plays an important role in viral inhibition (26). Given this and the results presented by Brestovitsky et al. (25), it is perhaps somewhat surprising that Forrester et al. demonstrated that infection of HeLa cells with Ad3, Ad4, Ad5, Ad7, Ad9, Ad11, and Ad12 all resulted in an increase in KAP1 phosphorylation, a marker of ATM activation. These results suggest that all viral serotypes investigated caused activation of ATM, however the kinetics of this varied between different serotypes (18).

This discrepancy can be explained by allowing for two different mechanisms of ATM activation secondary to Ad5 infection. The first is proposed to be localized MRN-mediated ATM activation associated with blocking of viral genome replication at the earliest stages of the viral life cycle, prior to MRN inhibition by viral proteins. The second is an MRN-independent, global ATM activation that occurs subsequent to viral genome replication and does not impact on viral replication (27). Thus, the increase in KAP1 phosphorylation representative of increased ATM activity would not be necessarily associated with a direct impact on viral replication.

#### The Effects of Virus Proteins on ATR

ATR is traditionally associated with recognition of single-stranded DNA (28). It would initially seem counter-intuitive for DNA viruses to have developed mechanisms of ATR interaction, nonetheless there is evidence that this is the case with adenovirus (25, 29, 30). It is frequently seen that infection with Ad5 leads to widespread histone 2AX phosphorylation (γH2AX) (31, 32). γH2AX is usually a marker for double-strand DNA breaks; however following infection, it can be seen throughout the nucleus. Nichols et al. demonstrated that, in adenovirus-infected cells, the greatest decrease in phosphorylation of γH2AX following inhibition of ATM, ATR, or DNA-PK was seen following ATR inhibition, suggesting that ATR is the primary mediator of adenovirus-induced H2AX phosphorylation. Large amounts of γH2AX phosphorylation were seen after the onset of viral genome replication and some γH2AX of a differing pattern was seen following input of viral DNA at high multiplicities (31). This suggests that there is a cell response to the presence of viral DNA and potentially a mechanism by which there is ATR stimulation in response to viral replication. As ATR is known to respond to a wide range of cellular stresses (33), it may be that viral replication is a sufficient cell stressor to cause this. Alternatively, it has been demonstrated that a significant number of single-stranded DNA sequences are generated during viral replication (34), the virus may, therefore, have developed a mechanism to interact with ATR to prevent this stage of the viral replication cycle from causing cell cycle arrest. There are a number of interactions between adenoviral proteins and the cellular ATR pathway that have been demonstrated.

E1b-AP5 (Ad E1b55K-associated protein 5) is a cellular protein that binds E1b55K in both Ad5-infected and Ad5-transformed cells (35). Blackford et al. have demonstrated that, in non-infected cells, this protein is localized to the nucleus but excluded from nucleoli. In the context of infection with adenovirus serotype 5 and 12, however, E1b-AP5 levels increase and it is redistributed to viral replication centers where is colocalizes with RPA32. Moreover, infection with these virus serotypes also seemed to lead to relocalization of ATR-interacting protein (ATRIP) to viral replication centers and colocalization with RPA. The two different viral serotypes appeared to have different effects on ATR kinase substrates. Whereas Ad12 infection was associated with marked E1b-AP5 and ATR-dependent hyperphosphorylation of RPA32 as well as hyperphosphorylation of Rad9, Ad5 infection did not have this effect, adding evidence that different virus serotypes have varying effects on the cell’s DNA damage response pathway (36). Forrester et al. have provided further evidence in support of this. The effect on checkpoint kinase 1 (Chk1) phosphorylation, an ATR substrate, in response to infection with different virus serotypes was investigated. The authors demonstrated that the overall effect on phosphorylation levels, as well as the timeline over which changes took place, differed markedly between serotypes (18). In addition, the ATR-activator protein topoisomerase-IIβ-binding protein 1 (TOPBP1) was degraded following infection with Ad12, but not with other viral serotypes investigated (18, 30).

Carson et al. provide evidence that the inhibition of ATR function by Ad5 is through effects on MRN. Cell lines with hypomorphic mutations in either Nbs1 or MRE11 were infected with either wild-type or E4-deleted Ad5. Results were compared when these cell lines were transduced with Nbs1 or MRE11 wild-type cDNA. There was decreased phosphorylation of the ATR substrates Chk1 and RPA32, suggesting decreased ATR function, when cells infected with E4-deleted Ad5 did not express wild-type Nbs1 or MRE11. Furthermore, infection of HeLa cells with an E4-deleted Ad5 mutant virus lead to phosphorylation of Chk1 and RPA32. This was markedly decreased in cells infected with Ad5 expressing E4orf3, but not in cells infected with Ad5 expressing E4orf6 or E1b55K. The authors did not find this effect on ATM signaling. Interestingly, when HeLa cells were infected with Ad5 mutants lacking E4, transfection with an Ad5 E4orf3 expression vector was sufficient to lead to a decrease in phosphorylation of Chk1 and RPA32. This was not, however, the case following transfection with an Ad12 E4orf3 expression vector or with an Ad5 E4orf3 expression vector mutated to abrogate the protein’s ability to mislocalize MRN. Ultimately, the authors provided evidence that MRN is key in the hyperphosphorylation of ATR substrates following infection with Ad5 lacking E4, and that this can be abrogated through expression of Ad5 E4orf3 and subsequent mislocalization of the MRN complex (29).

### The Effects of Virus Proteins on p53

The full range of p53’s effects within the cell is still being elucidated; however, it is clear that p53 has an inherent role in the cell’s response to stressors and DNA damage (37, 38). It is, therefore, perhaps no surprise that its function is targeted by adenoviruses, though the exact effects of this targeting appear to vary between serotypes. Ad5 E4orf3 and E4orf6 are known to have an effect on p53 through interaction with the E1b55K protein (39, 40). Liu et al. have demonstrated that Ad5 E1b55K co-localizes with p53 to aggresomes. Following transfection of 293 cells with E4orf6, the majority of cells demonstrated p53 depletion, and in those cells where p53 was visible, it was associated with E4orf6 in aggresomes (22). Harada et al. used a proteomics-based approach to demonstrate that the E1b55K–E4orf6 complex interacts with a number of cellular proteins to lead to the polyubiquitination of p53 *in vitro* (41). It is thought that E1b55K–E4orf6, therefore, leads to p53 depletion through ubiquitin-dependent proteasome-mediated destruction.

The degradation of p53 does not appear to be conserved across all virus serotypes tested. Forrester et al. have demonstrated that, though infection with Ad4, Ad5, and Ad12 leads to p53 degradation, this does not occur following infection with Ad3, Ad7, Ad9, and Ad11. Interestingly, there appeared to be, in fact, a marked increase in p53 levels in cells infected with Ad3, Ad7, and Ad11. The authors present evidence that this is, however, not transcriptionally active. It was shown that MDM2 levels were decreased following infection with all virus serotypes investigated, including those associated with increasing levels of p53. Furthermore, following Ad3 and Ad7 infection of cells containing a p53 plasmid and a luciferase reporter construct, though there was a significant increase in p53 levels, there was only a minor increase in reporter transcription. In addition, cells infected with these virus serotypes demonstrated decreased protein levels of p21, a p53-regulated gene, secondary to decreased levels of p21 mRNA (18).

It remains to be seen how this variable interaction between p53 and the different adenovirus serotypes will impact on a potential synergistic effect with radiotherapy. The role of p53 within the cell is a complex and far-reaching one, hence its moniker “the guardian of the genome” (42). In particular, p53 inactivation is key in oncogenesis and it is mutated in approximately 50% of all cancers (43). Its function as a key modulator of apoptosis is, therefore, in balance with that of a DNA damage responder. Wild-type p53 is associated with enhanced chemosensitivity and radiosensitivity, but the exact p53-mediated cellular response to these stressors appears to be reliant on a range of cellular factors (44). As such, given the complex interactions between adenovirus infection and the cellular stress response, it is difficult to predict which p53 transcriptional pathway will ultimately be most affected by combination therapy.

### Effects of Virus on DNA Repair Proteins

#### Effects of Virus Proteins on NHEJ

A physical interaction between an oncolytic viral protein and a protein heavily involved in the DNA damage response was first demonstrated by Boyer et al (45). Glioma cells with or without DNA PK were infected with either wild-type Ad5 or a mutant lacking E4. Concatemers formed in cells expressing DNA PK when these were infected by mutant virus, but not wild-type. Interestingly, no concatemerization was seen in cells lacking DNA PK, and following plasmid transfection of 293 cells with plasmids expressing DNA PK and either E4orf6 or E4orf3, there appeared to be co-immunoprecipitation of DNA PK and these viral proteins.

Since this time, the major effect of multiple virus serotypes on NHEJ has been demonstrated to be through degradation of DNA ligase IV. This was initially shown in Ad5 by Baker et al., who demonstrated that degradation was dependent on expression of the E1b55K–E4orf6 complex and was likely mediated by ubiquitination and subsequent targeting by the proteasome (46).

Degradation of DNA ligase IV has also been observed post infection with adenovirus serotypes 3, 4, 5, 7, 9, 11, and 12 (18, 30). Crucially, it was the only cellular protein degraded by all adenoviral serotypes tested by Forrester et al., suggesting that it plays a key role in the inhibition of the DNA damage response pathway by human adenoviruses (18).

#### Effects of Virus Proteins on HR

There is currently limited evidence on the effect of oncolytic viruses on HR. Tookman et al. investigated the impact of HR status in ovarian cancer on adenovirus infection. Two cell lines were utilized, both from the same ovarian cancer patient. The first was obtained during a platinum-sensitive relapse, contained a deleterious BRCA2 mutation and was, therefore, HR defective, the second was obtained during a subsequent platinum-resistant relapse following development of a secondary BRCA2 mutation which restored the open reading frame and had, therefore, regained HR function. Cells were infected with wild-type Ad5, Ad11 or Ad35, or one of two Ad5 mutants, both containing an E3b region deletion, one of these also containing a deletion in the region of E1A CR2. Interestingly, the authors found that the cell line with functional HR demonstrated a significant decrease in cell survival following infection by all three Ad5 viruses when compared to the cell line with defective HR. Confocal microscopy showed colocalization between viral replication centres (VRC) and BRCA2. Surprisingly, the authors also noted colocalization of VRC and Rad51 foci in both the presence and absence of BRCA2, and this was confirmed in a number of other cell lines. This is the first demonstration of recruitment of HR proteins to the adenovirus VRC. Co-immunoprecipitation was consistent with an interaction between Rad51 and Ad5 E2 DNA binding protein. Furthermore, depletion of Rad51 resulted in reduced cytotoxicity and viral replication following infection with the above-mentioned Ad5 mutants, BRCA2 depletion likewise leads to reduced cytotoxicity (47). These data suggest these HR proteins are utilized by Ad5 to improve viral replication and cytotoxicity.

## Adenovirus as a Radiosensitizer

Adenoviruses have developed a range of interactions with cellular DNA damage repair proteins to allow successful viral replication. This has implications for the initiation of a number of DNA repair pathways activated in response to radiation-induced damage, in particular all adenoviral serotypes tested appear to target NHEJ repair (18). The hypothesis that oncolytic adenovirus infection would work synergistically with radiotherapy has been tested by a number of groups. The combination of CG7870 with radiation resulted in a synergistic increase in cell killing, both *in vitro* and *in vivo* in the LINPAC xenograft model, than either agent alone (48). Toth et al. studied 3 Ad5-based vectors and combined them with radiation in A549 lung cancer cells (49). Again they found that *in vivo* and *in vitro* tumor cell kill was increased with the combination approach than was seen with either agent alone. Similar findings have been noted with a variety of different adenoviral vectors in other cell types, including ovarian cancer cell lines (50) and glioma xenografts (51). Importantly, however, the effect of radiosensitization does not appear to extend to normal tissues. The combination of Ad5/CMV/p53 radiosensitized two non-small cell lung cancer cell lines (A549 and H322) *in vitro* and in xenograft models, in a synergistic fashion, but did not show an increased radiosensitization effect on normal lung fibroblasts (52). These observations provide a framework to consider the clinical rationale for adenovirus and radiation combinations, as discussed in section “Clinical Efficacy and Toxicity.”

Although radiation damage to the viral genome could render the particle inactive, the small size of the genome is, statistically, unlikely to be affected by standard X-ray photons. X-rays are sparsely ionizing meaning that primary ionization events are well separated, at least microscopically. In comparison to the human genome (3 × 10^9^ bp), the relative size of the genome for most therapeutically employed viruses (adenovirus is about 3.5 × 10^3^ bp) means the ionization events are unlikely to trouble the majority of particles. The ability to influence ATR and by extension single-strand break repair, which is a far more common type of cellular response to radiation, is also beneficial. Single-strand breaks are easily repaired avoiding cell death. The arrest of single-strand break repair increases the likelihood of future catastrophic damage and apoptotic death.

## Radiotherapy and the Immune Response

### Immune Inhibition versus Immune Stimulation

The interplay between immunostimulatory and immunoinhibitory pathways in response to radiation is a complex and intricate one (53–55). Radiotherapy has long been thought to elicit an immunosuppressive effect (56, 57). A number of cell types have been implicated in this. Regulatory T cells have been shown to play an important role in the inhibition of an antitumor immune response following radiotherapy (58). There is also evidence for immunoinhibitory roles played by, and potential increased influx resulting from radiotherapy, of both tumor-associated macrophages (59, 60) and myeloid-derived suppressor cells (60, 61). Furthermore, radiation-induced effects on dendritic cells have been demonstrated *in vitro* to shift cytokine release away from activation and toward tolerization (62). Effects on different cell types and the tumor microenvironment have recently been elegantly reviewed elsewhere and an in-depth discussion is outwith the scope of this article (53, 55, 60).

On the other hand, the body of literature presents us with multiple preclinical and clinical examples of ways in which the host antitumor immune response can actually be augmented by radiotherapy (63–71). A number of mechanisms have been proposed as mediating this. These include preferential radiotherapy-mediated killing of radiosensitive suppressor T cells (63, 64), increased immune cell infiltration of tumors (65), activation of dendritic cells (66, 67), improved antigen presentation both in draining lymph nodes (65) and within the tumor itself (68), induction/upregulation of cell surface markers that interact with cytotoxic T lymphocytes (69, 72), release of immunostimulatory molecules subsequent to radiation-induced immunogenic cell death (70, 71, 73), and the production of pro-inflammatory cytokines and chemokines (73).

We are still unable to predict which way the fine balance will tip in response to tumor irradiation. Interestingly, it has been proposed that ablative radiotherapy (typically greater than 6 Gy per fraction) favors a T-cell-dependent immunostimulatory antitumor response when compared to more traditional fractionation schedules (1.8–2 Gy per fraction), which may favor an immunosuppressive response (66, 68, 74, 75), though Dewan et al. have presented data that would appear to contradict this (76). The ideal fractionation schedule favoring immune stimulation has yet to be determined and is likely to be influenced by a large number of host and tumor-specific variables (72). Currently, focus is shifting toward combining ionizing radiation with immunotherapy to tip the scales in favor of promoting an antitumor immune response (54). In particular, a number of studies are currently planned or ongoing focusing on combining radiation with checkpoint inhibitors (53).

### The Abscopal Effect

The abscopal effect, a phenomenon whereby ionizing radiation of a tumor leads to reduction of tumor growth outwith the field of radiation, was initially described by Mole (77). Demaria et al. demonstrated that it is at least partially immune mediated (78). Though a number of clinical cases describing the abscopal effect have now been documented, this phenomenon is by no means common (75, 79). With the advent of combination radiotherapy with checkpoint inhibitors, it is hoped and anticipated that cases demonstrating beneficial abscopal effect will become more common in the coming years.

## Oncolytic Adenoviruses and the Immune Response

Viruses utilize a number of mechanisms to evade the normal immune response. It has been proposed that the aberrant intracellular pathways in cancer cells, however, make them particularly vulnerable to targeting by oncolytic viruses for destruction via a mechanism of immunogenic cell death (10). This would provide an alternative means by which the viruses may act synergistically with radiotherapy and tip the scales in favor of immunostimulation. One potential mechanism to enhance this further is the development of armed oncolytic viruses that express immunostimulatory molecules (produced locally by virus-infected tumor cells and released into the tumor microenvironment), providing further signals that can enhance an antitumor immune response.

### Oncolytic Adenovirus Mechanisms of Immune Stimulation

The different mechanisms of immunogenic cell death have recently been elegantly reviewed (80). With regard to cellular responses to invasion of pathogens, microorganism-associated molecular patterns (MAMPs)/pathogen-associated molecular patterns (PAMPs) interact with intra- and extracellular pattern recognition receptors, such as toll-like receptors and nucleotide-binding oligomerization domain-like receptors. In response to viral infection, a subsequent danger response is elicited and is associated with formation of inflammasome complexes and secretion of type I interferons. Pathogens, including viruses, demonstrate a range of mechanisms developed to avoid this immune detection and stimulation (80, 81).

In addition to PAMPs/MAMPs, virus-mediated cytotoxicity pathways may also be intrinsically immunogenic via damage-associated molecular patterns. For example, Dyer et al. have recently published data on the ability of Enadenotucirev (EnAd), a chimeric group B adenovirus currently undergoing clinical trials, to induce proinflammatory cell death (82). Mode of cytotoxicity displayed features consistent with oncosis, and infection with EnAd led to release of the pro-inflammatory markers heat shock protein 70 (HSP70) and high-mobility group box-1 (HMGB1) from cells. The pro-phagocytic marker calreticulin was also increased on tumor cells infected with EnAd. Moreover, infection of tumor cells lead to a significantly higher level of dendritic cell-mediated T cell activation than wild-type Ad5. These data suggest that induction of immunogenic cell death may be an additional mechanism by which oncolytic viruses could work synergistically with radiotherapy.

### Arming Oncolytic Adenoviruses to Enhance Immune Stimulation

A number of different mechanisms have been utilized to “arm” oncolytic adenovirus and enhance the immune response. For example, Li et al. carried out a phase I dose escalation study investigating the effect of H103, a recombinant Ad2 virus overexpressing HSP70 (83). 27 patients with advanced solid tumors received intratumoral treatment. Clinical benefit rate (partial response, minor response, or stable disease) was 48.1%, with 11.1% experiencing partial response and, interestingly, three patients demonstrating transient regression of some distant non-injected areas of metastasis, though RECIST criteria for response were not met. The most frequently experienced side effects were local injection-site reaction and fever, hematological toxicities were observed in five patients.

A phase I study on the intravesical use of a GM-CSF expressing adenovirus, CG0070, in non-muscle invasive bladder cancer was carried out by Burke et al. (84). Of 35 patients treated, 17 had complete response (CR, 48.6%), with a median CR duration of 10.4 months. No clinically significant treatment-related toxicities were reported.

CGTG-602 (Ad5/3-E2F-Δ24-GMCSF) was another oncolytic adenovirus engineered to express GM-CSF (85). *In vivo* this virus demonstrated selective replication in tumor cells and appeared to induce an immune-mediated antitumor response. Intratumoral administration was carried out in 13 patients with advanced metastatic tumors. 6 were able to be assessed with PET CT, of these 83% demonstrated radiological disease control and PET response rate was 50%, including one patient who demonstrated complete metabolic response in a non-injected site. Tumor marker assessment indicated potential benefit in 6 out of 10 patients who had elevated markers at baseline.

Sova et al. present data on the use of a TNF-related apoptosis-inducing ligand (TRAIL) expressing adenovirus vector (86). This vector was able to induce tumor-specific apoptosis both *in vitro* and *in vivo*. In a mouse model for colorectal liver metastases, when compared to untreated controls, intravenous administration of the vector resulted in an approximately 10-fold tumor burden reduction versus approximately 1.5 to 3 fold in response to non-TRAIL expressing vectors, and complete eradication of metastases in three out of five mice. There was transient elevation of a liver enzyme following vector infusion but no histological changes of normal liver tissue.

Finally, Hirvinen et al. generated Ad5/3-D24-hTNFa, an oncolytic adenovirus expressing human TNFa and selective to retinoblastoma protein defective cells (87). Cell death caused by this virus *in vitro* was associated with a significant increase in ATP release compared to control virus, and increased, but not significant, levels of calreticulin exposure and HMGB1 release, thereby displaying some features of immunogenic cell death. *In vivo*, intratumoral injection resulted in significant tumor growth delay and prolonged survival compared to control virus. There was likewise significant reduction in tumor growth compared to control virus in a syngeneic mouse melanoma model. Interestingly, the authors investigated the combination of this virus with radiotherapy treatment *in vivo* and *in vitro*. Combination treatment had no impact on cell viability *in vitro. In vivo*, treatment with Ad5/3-D24-hTNFa combined with radiotherapy in a prostate cancer xenograft model led to a significant reduction in tumor growth when compared to treatment with mock or control virus and radiotherapy, but there was no difference between control virus and Ad5/3-D24-hTNFa in the immunocompetent mouse melanoma model, which the authors postulated may have been related to the inherent radioresistant nature of melanoma.

## Clinical Efficacy and Toxicity

To date, clinical experience with virus/radiation combinations has been limited to local (most commonly intratumoral) administration. This mode of delivery facilitates direct infection, ensuring correct dosing and avoiding the rapid hepatic uptake seen with systemic delivery (88). The downside is only tumor types that can be easily accessed with a needle, such as skin, head, and neck cancers or prostate cancers, are considered suitable for clinical trials. Nevertheless, the results of these studies provide us with useful mechanistic indicators as well as guiding assessment of toxicity. While the authors acknowledge that there are other oncolytic viruses in clinical practice, we will focus on the clinical experience with adenoviral agents.

A study of intraprostatic injection of an oncolytic Ad5 PSE/PBN E1A-AR (Ad5, adenovirus serotype 5; prostate-specific enhancer; PBN, rat probasin promoter; E1A, early region 1A; androgen receptor), combined with either low or high dose rate radiation therapy, showed remarkably few side effects (89). Although DNA damage, as assessed by γH2AX foci, viral replication and viral induced cell death all favored the high-dose radiation arm, the side effect profile was similar in both arms. This indicates that the therapeutic efficacy is separate from the toxicity, in contrast to traditional radiosensitizers where a higher dose often increases both efficacy and toxicity. These findings support the large body of preclinical data that there is little additive toxicity to that seen with either agent alone (90).

A phase I trial of intraprostatic injection of a replication-competent adenovirus in combination with radical dose (74 Gy delivered in daily 2 Gy fractions) of intensity modulated radiotherapy (IMRT) showed no significant differences in gastro-intestinal or genitourinary toxicity in comparison to the toxicity seen when administering the adenovirus as a single agent (91). The investigational agent had already proven safe and efficacious as a single agent (92). These results were confirmed in a follow on randomized phase II trial (93). There was a non-significant 42% reduction in biopsy positivity in the investigational arm, suggesting improved efficacy and synergy with radiation. Clinical outcomes at 2 years show no difference, likely reflecting the excellent prognosis of both groups. A phase II/III (ReCAP) open label adaptive trial of 280 men, randomized to combination treatment or radiation efficacy, with biochemical failure free survival as the primary endpoint (94). Other groups have shown that administering a different type of adenovirus is safe, both concurrently and after radiation to the prostate (95, 96), when all cells should be at maximal damage and repair rates. Again, the viral compound was administered intratumorally.

There is also evidence from early phase clinical trials that a combination approach of radical dose (76 Gy delivered in 2.17 Gy daily fractions) with Ad5 replication defective adenoviral vector stimulates a systemic response (97). IMRT was commenced 48 h after the second of three doses of the viral agent, therefore, patients were effectively loaded and then treated concurrently. Again, drug was administered intraprostatically. Both HLA DR+ CD8+ and CD4+ T cells were increased in the combination arm compared to the radiation alone arm, suggesting the potential development of a Th1 response.

In a mixed solid tumor cohort, an adenovirus vector under the control of EGF-1 promoter was combined with radiation in 36 patients (98). 70% of subjects showed evidence of a partial response, with the main side effects relating to intratumoral administration of the agent. Using the same agent in combination with chemoradiation for squamous cell carcinomas of the head and neck a phase I dose escalation trial was performed (99), again with intratumoral administration. The main dose limiting toxicity (DLT) seen was thrombosis, with no increase in acute radiation side effect incidence or intensity, underlining the safety of the combination approach. Locoregional response was 83.3%. Preclinical studies with this agent have shown impressive ability to suppress regional metastatic node formation highlighting its ability to influence intrinsic tumor biology (100). Incrementally increasing doses of the same agent were also combined with radiation in soft tissue extremity sarcoma (101). No DLT was noted and the combination was well tolerated. 91% of patients undergoing surgery showed a pathological CR to treatment highlighting significant potential synergy between both agents. The same adenovirus composite has been successfully combined with radical chemoradiotherapy (50.4 Gy delivered in 1.8 Gy daily fractions concurrently with fluoropyrimidines) for locally advanced pancreatic cancer in a non-randomized phase I/II setting (102). The main DLTs were pancreatitis and cholangitis but no specific increases in observed acute radiotherapy or chemotherapy side effects, respectively, were seen. The adenovirus was administered intratumorally.

Combination of yet another adenovirus, designed to transfer p53 to malignant cells, in a radically treated non-small cell lung cancer population has shown impressive response data (103). This prospective phase II trial of 60 Gy in combination showed no evidence of pathologically viable tumor in 63% of patients (12 out of 19) evaluable. The most common adverse events were virus related; fevers (79%), and chills (53%).

Ongoing studies in brain malignancies, such as glioblastoma multiforme (GBM), are also encouraging. Intratumoral injection at the time of surgery of an adenoviral vector expressing HSV thymidine kinase gene, combined with radical chemoradiation post operatively (104), has been tested prospectively. 12 of 13 patients completed therapy, at varying dose levels in this phase Ib trial, with no DLTs or significant toxicity. A phase II trial is ongoing (NCT00589875). Further evidence of safety in GBM patients is provided by the small phase I study that used a conditionally replicating HSV, G207 (105). Following two prior safety studies with single-agent use, they showed, in nine patients, that intratumoral injection followed by 5 Gy of radiation 24 h later had no increased risk of toxicity. Preclinical data with G207 also points to efficacy in other tumor sites, such as head and neck SCC and lung cancer (106, 107) (see above).

Taken together, these clinical data support the safety of a combination approach of radiation with a range of adenovirus constructs. The most commonly reported adverse events are related to the local administration and investigational agent itself rather than any increase in expected normal tissue toxicity mediated by excessive radiosensitization. Although clinical trials have not yet progressed to the point of assessing efficacy as an endpoint, several are in conduct. The optimum timing and sequencing of the two modalities has yet to be decided. So far concurrent delivery has been most used, appears safe and effective with no negative effects on viral biology. Further mechanistic work on sequencing is required, however. The need to access the tumor directly has limited the scope of clinical investigation, both in terms of tumor type and tumor stage. The future of oncolytic viral therapy really lies in newer agents with the ability to be delivered systemically (9, 108). This approach would not only allow the treatment of many more tumor sites but also, potentially, target micrometastatic disease such as nodal spread. The ability to successfully access tumors remotely will offer novel opportunities to further understand the reciprocal biology and elucidate the optimal approach to combination therapy.

## Future Directions

Oncolytic adenoviruses have developed several mechanisms to inhibit cellular DNA damage repair pathways. This creates a logical case for additive and potentially synergistic efficacy when oncolytic viruses are combined with radiotherapy, although the outcome may also depend on the radiotherapy protocol employed. Not only do these viruses promise activity as cancer-selective radiosensitizers, the observation that some oncolytic adenoviruses naturally mediate an immunogenic cell death mechanism provides the possibility of provoking an anticancer immune response. Coupled with the option to express therapeutic proteins selectively at the tumor site, this creates an appealing and potentially unique targeted immunostimulatory strategy that builds on the strengths of both component approaches. Accordingly, we anticipate the development of systemically administered viruses designed as cancer-targeted radiosensitizers, capable of stimulating potent anticancer immune responses to stimulate activity against tumor deposits both inside and outside the field of irradiation. Such an approach should be perfectly complementary to the current generation of checkpoint inhibitors, and it is feasible to anticipate a further increase in utility if all three approaches are combined in future treatment strategies.

## Author Contributions

All authors listed have made a substantial, direct and intellectual contribution to the work, and approved it for publication.

## Conflict of Interest Statement

LS and KF own equity in Psioxus Therapeutics Ltd., which is developing an oncolytic adenovirus for treatment of cancer. All other authors declare no conflict of interest.
